# Supplementary material for: Oligomerization of the Clostridioides difficile Transferase B Component Proceeds through a Stepwise Mechanism
Source: bioRxiv. 2025 May 6:2025.05.06.652354. Preprint. [Version 1] doi: 10.1101/2025.05.06.652354 (PMC12248058; doi:10.1101/2025.05.06.652354)
Supplement: Supplement 1 [file NIHPP2025.05.06.652354v1-supplement-1.pdf]

bioRxiv preprint doi: https://doi.org/10.1101/2025.05.06.652354; this version posted May 6, 2025. The copyright holder for this preprint (which was not certified by peer review) is the author/funder, who has granted bioRxiv a license to display the preprint in perpetuity. It is made available under aCC-BY 4.0 International license.

SUPPLEMENTAL INFORMATION

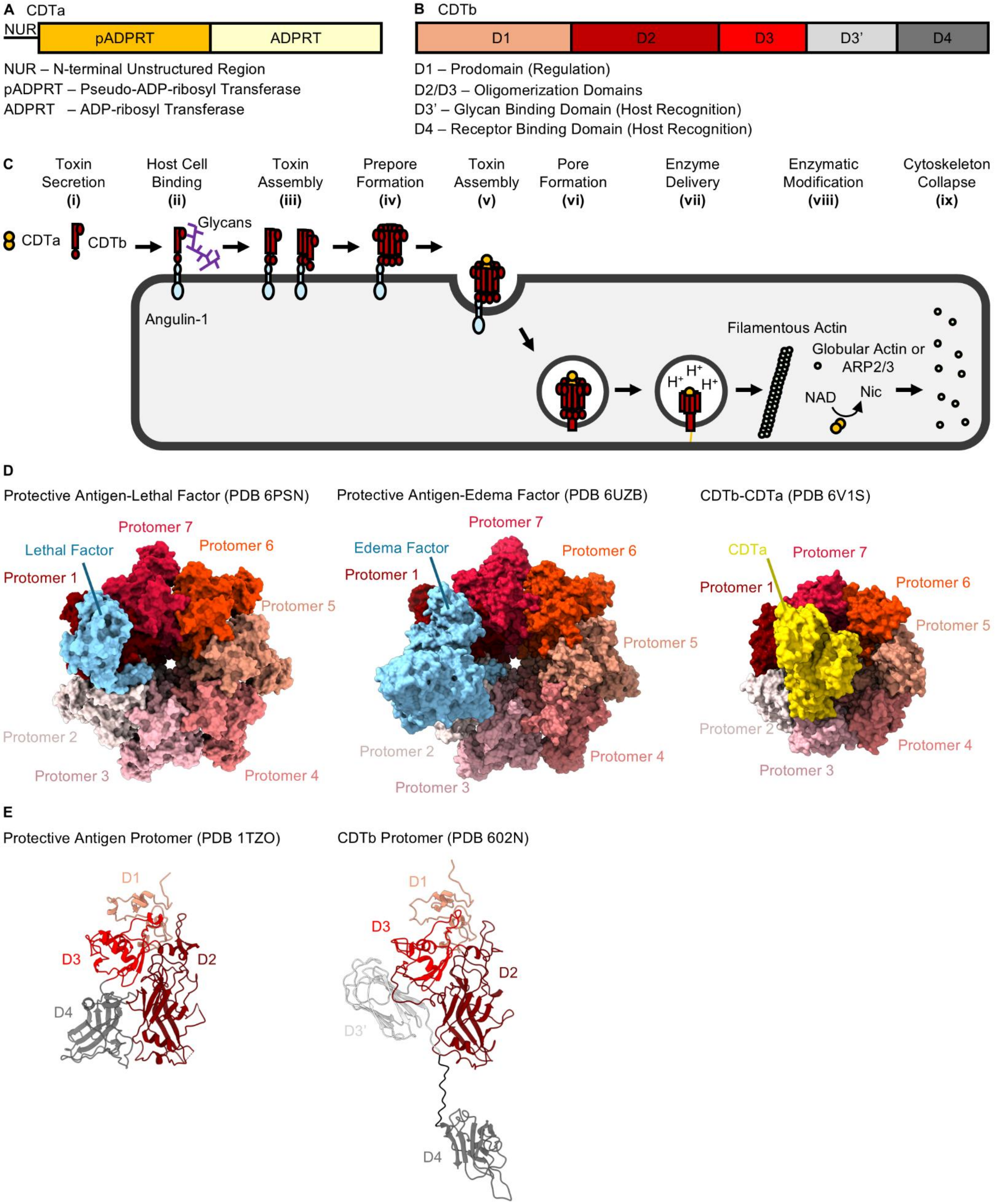

bioRxiv preprint doi: https://doi.org/10.1101/2025.05.06.652354; this version posted May 6, 2025. The copyright holder for this preprint (which was not certified by peer review) is the author/funder, who has granted bioRxiv a license to display the preprint in perpetuity. It is made available under aCC-BY 4.0 International license.

Supporting Fig 1 – The Structure and Proposed Mechanism of CDT Intoxication. The previously defined domain organization of CDTa and CDTb are depicted in panels (**A**) and (**B**), respectively. (**C**) The accepted model of CDT intoxication begins with toxin secretion at the site of infection (i). Once secreted, CDTb localizes to host cell through a receptor known as Angulin-1. CDTb has also been shown to interact with glycans though it is not clear what role this interaction plays during intoxication (ii). CDTb is then proteolyzed and oligomerizes (iii) to form a structure referred to as the ‘prepore’ (iv). The prepore binds a single copy of CDTa and enters cells via endocytosis (v). Within the endosome CDTb undergoes a structural rearrangement leading to the formation of a membrane-spanning channel or ‘pore’ (vi). In response to the environment of the maturing endosome, CDTa passes through the CDTb pore and into the host cell cytoplasm (vii). Inside the cell, CDTa modifies globular actin and the actin related protein 2/3 (Arp2/3, viii) leading to cytoskeletal collapse and a cell rounding phenotype (ix).

bioRxiv preprint doi: https://doi.org/10.1101/2025.05.06.652354; this version posted May 6, 2025. The copyright holder for this preprint (which was not certified by peer review) is the author/funder, who has granted bioRxiv a license to display the preprint in perpetuity. It is made available under aCC-BY 4.0 International license.

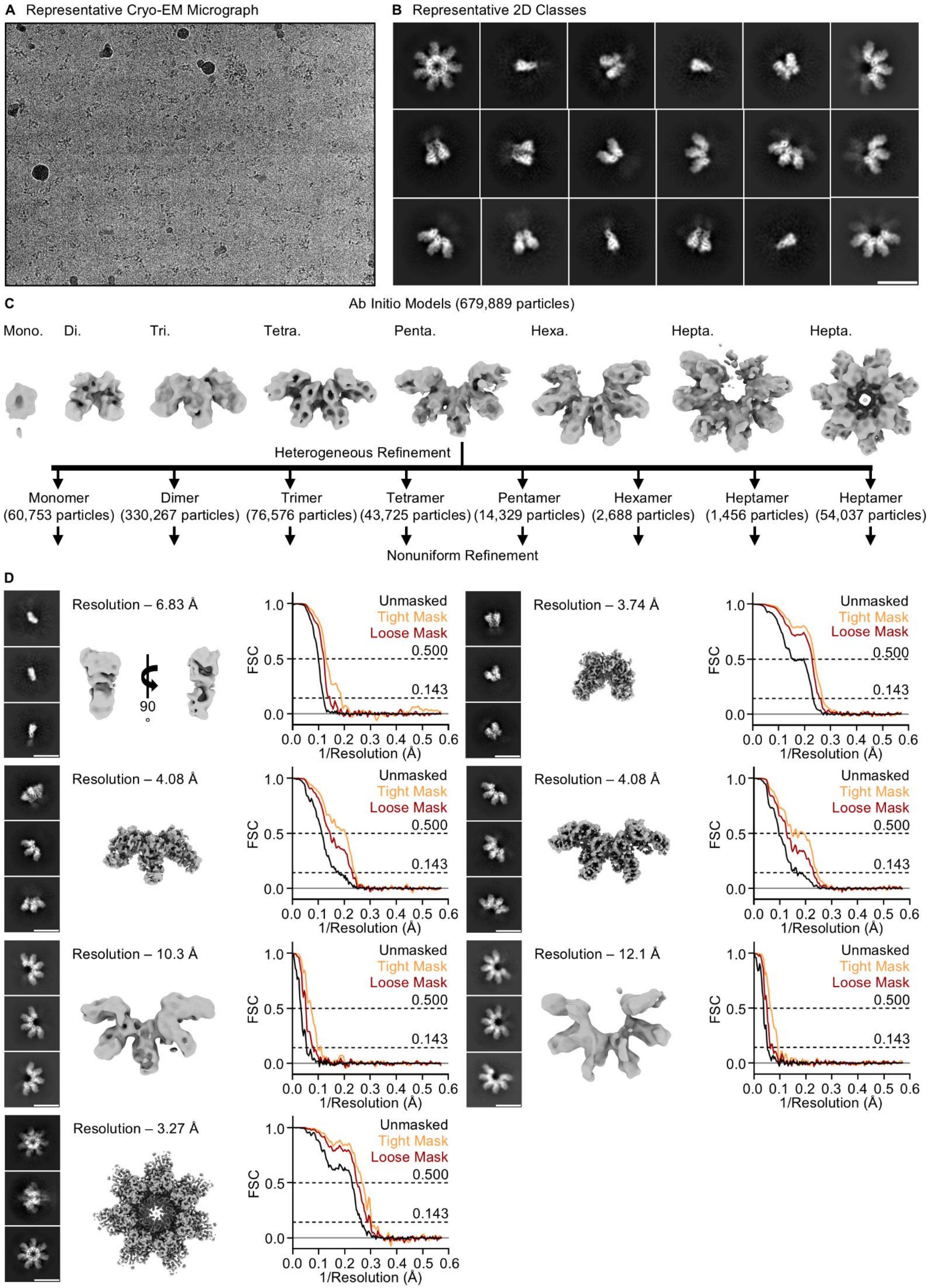

bioRxiv preprint doi: https://doi.org/10.1101/2025.05.06.652354; this version posted May 6, 2025. The copyright holder for this preprint (which was not certified by peer review) is the author/funder, who has granted bioRxiv a license to display the preprint in perpetuity. It is made available under aCC-BY 4.0 International license.

Supporting Fig 2 – Cryo-EM Classification of CDTb Oligomeric Intermediates. (**A**) A representative micrograph collected of the sample containing CDTb oligomeric intermediates. (**B**) Two-dimensional classification was used to sort particles into distinct oligomeric states. These particles were then used to generate eight ab initio models (**C**). The ab initio models were refined to generate high resolution maps to be used as references for further refinement. All maps that were generated are shown below with representative two-dimensional classes illustrated on the left. Fourier shell correlation plots were used to estimate the global resolution of each map using a 0.143 cutoff as shown to the right of each map.

bioRxiv preprint doi: https://doi.org/10.1101/2025.05.06.652354; this version posted May 6, 2025. The copyright holder for this preprint (which was not certified by peer review) is the author/funder, who has granted bioRxiv a license to display the preprint in perpetuity. It is made available under aCC-BY 4.0 International license.

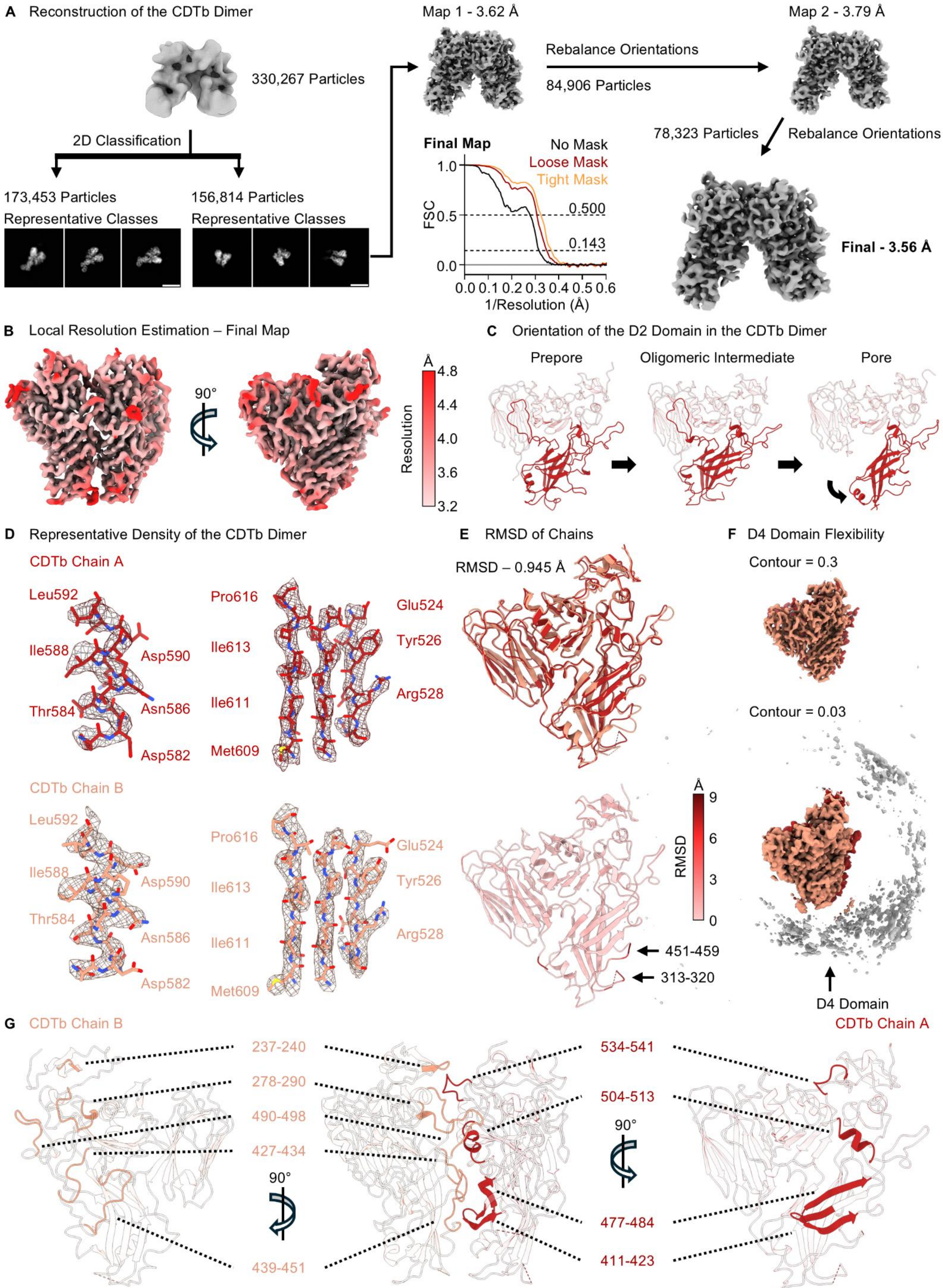

bioRxiv preprint doi: https://doi.org/10.1101/2025.05.06.652354; this version posted May 6, 2025. The copyright holder for this preprint (which was not certified by peer review) is the author/funder, who has granted bioRxiv a license to display the preprint in perpetuity. It is made available under aCC-BY 4.0 International license.

Supporting Fig 3 – Reconstruction and Structure of the CDTb Dimeric Assembly Intermediate. (**A**) Particles corresponding to the CDTb dimer were subjected to a final round of two-dimensional classification to separate structurally distinct particles. This dataset was then used to reconstruct a map of the CDTb dimeric assembly intermediate in three-dimensional space to a global resolution of 3.62 Å. Due to issues arising from preferred orientation, the resulting map suffered from anisotropic resolution and was subjected to iterative jobs to rebalance particle orientations within the dataset. The final map was reconstructed to a resolution of 3.56 Å as determined by Fourier shell correlation using a 0.143 cutoff. (**B**) The local resolution of the CDTb dimer is displayed on the final map. (**C**) The CDTb dimeric assembly intermediate is observed in a prepore-like configuration wherein the D2 domain adopts an outward facing conformation as opposed to the inward facing conformation reported in the structure of the CDTb pore. (**D**) Representative density of Chains A and B in the CDTb dimeric intermediate. (**E**) An overlay of Chains A and B is shown at the top with the backbone RMSD of all residues observed in both structures indicated at the bottom. (**F**) A low contour map of the CDTb dimer illustrating a lack of density corresponding to the CDTb D4 receptor binding domain (top). A high contour rendering of the same map indicates the presence of density that can be attributed to the D4 domain (bottom). (**G**) Residues involved in the interface that facilitates CDTb dimerization. Chain A is shown in scarlet and Chain B in salmon.

bioRxiv preprint doi: https://doi.org/10.1101/2025.05.06.652354; this version posted May 6, 2025. The copyright holder for this preprint (which was not certified by peer review) is the author/funder, who has granted bioRxiv a license to display the preprint in perpetuity. It is made available under aCC-BY 4.0 International license.

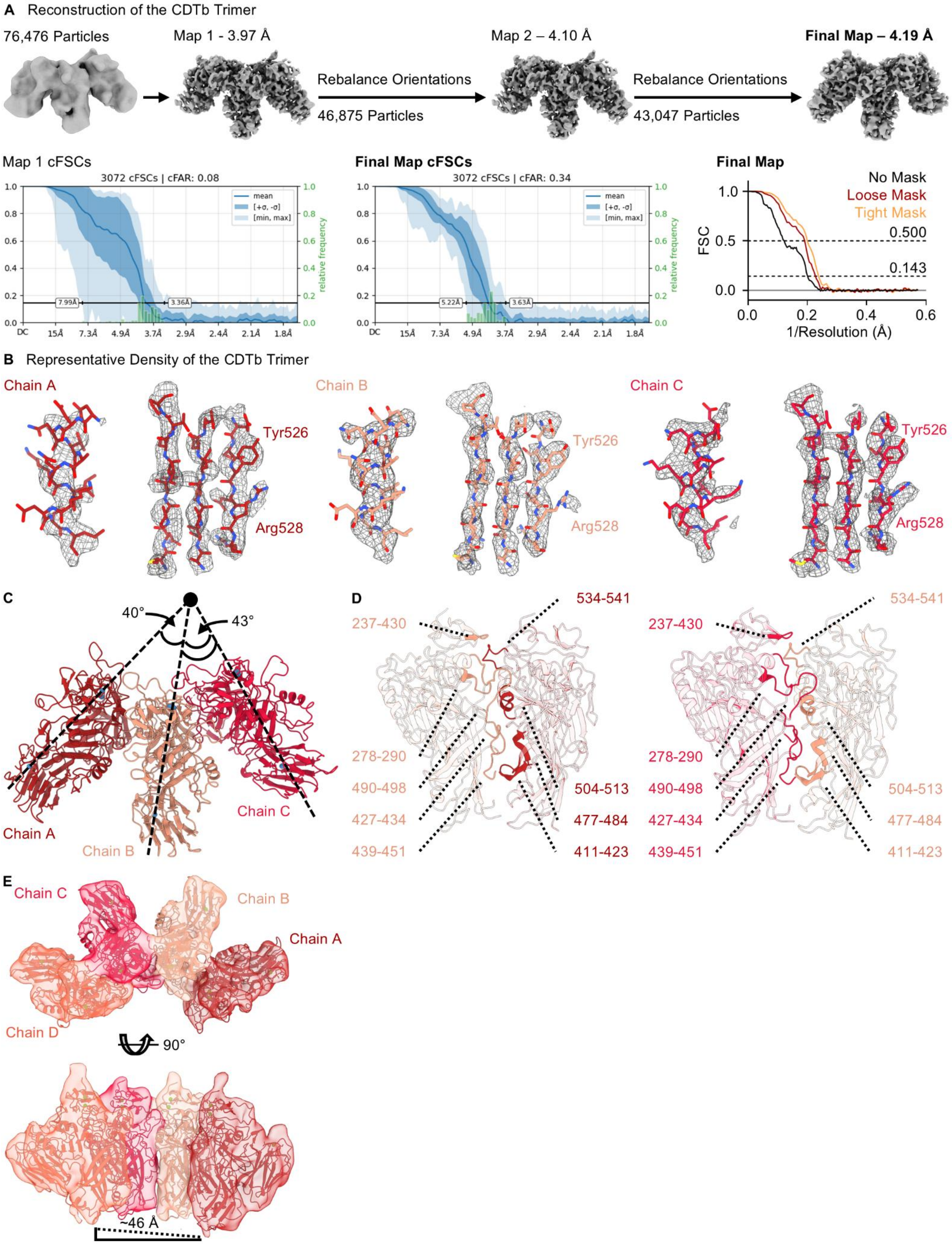

bioRxiv preprint doi: https://doi.org/10.1101/2025.05.06.652354; this version posted May 6, 2025. The copyright holder for this preprint (which was not certified by peer review) is the author/funder, who has granted bioRxiv a license to display the preprint in perpetuity. It is made available under aCC-BY 4.0 International license.

Supporting Fig 4 – Reconstruction and Analysis of the CDTb Trimeric and Tetrameric Assembly Intermediates. (**A**) An initial map of the CDTb trimeric assembly intermediate was reconstructed to 3.97 Å resolution. Particles were rebalanced iteratively to limit the effect of preferred orientation for this sample resulting in a final map of 4.19 Å resolution. (**B**) Representative density of the CDTb trimeric intermediate with the constructed model fit into the density. Chain A is depicted in scarlet, Chain B in salmon, and Chain C in red. (**C**) The relative orientations of Chains A and B and Chains B and C with respect to the central symmetry axis of the symmetric heptamer. (**D**) Residues facilitating interactions between Chains A and B (left) and Chains B and C (right). (**E**) The low-resolution map that was reconstructed of the CDTb tetrameric assembly intermediate. Chain A is shown in scarlet, Chain B in salmon, Chain C in red, and Chain D in peach.

bioRxiv preprint doi: https://doi.org/10.1101/2025.05.06.652354; this version posted May 6, 2025. The copyright holder for this preprint (which was not certified by peer review) is the author/funder, who has granted bioRxiv a license to display the preprint in perpetuity. It is made available under aCC-BY 4.0 International license.

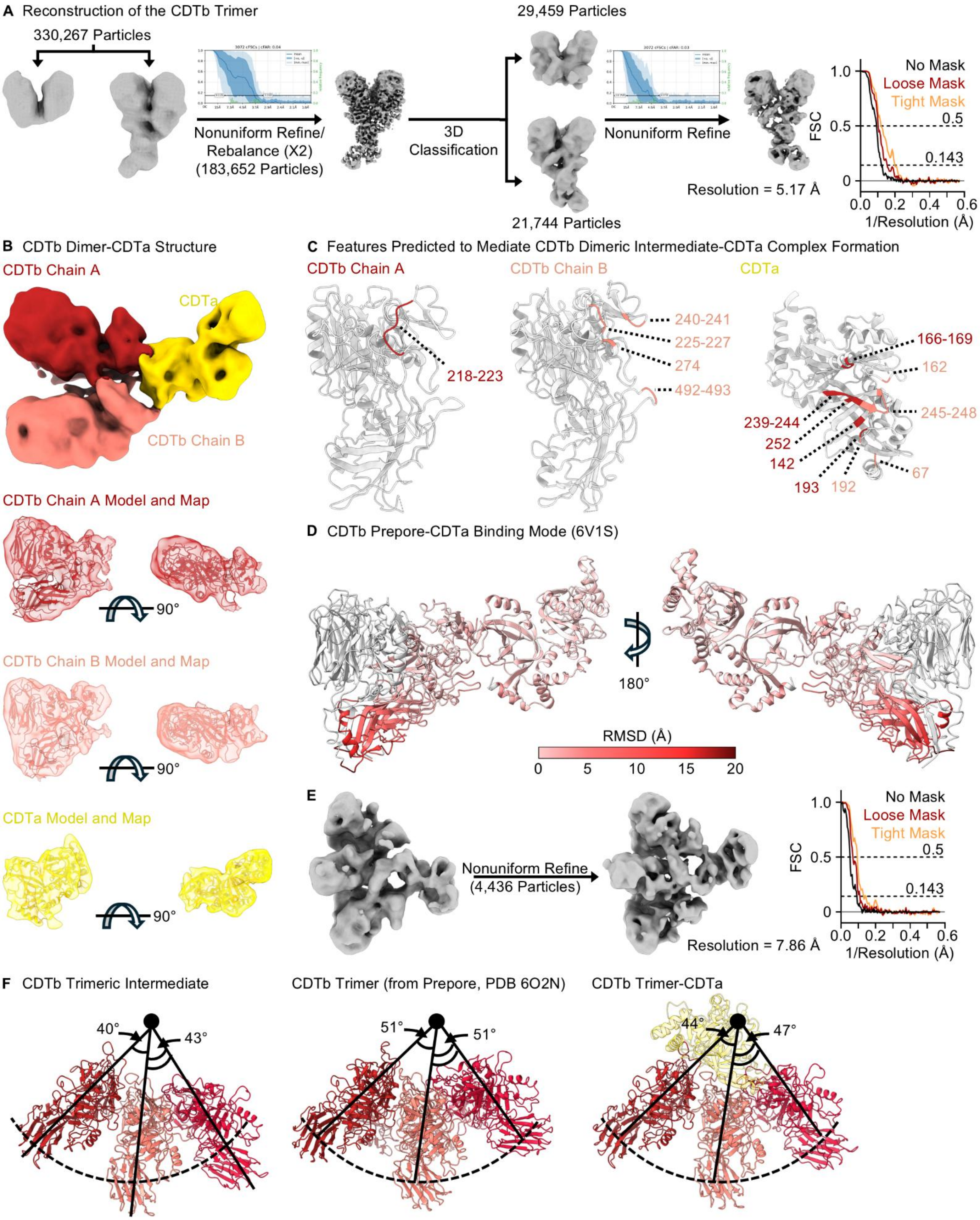

Supporting Fig 5 – CDTa Bound Oligomeric Intermediates. (**A**) Reconstruction of the CDTb dimeric intermediate bound to CDTa resulted in a map with a global resolution of 5.17 Å. Conical FSCs (inset) were used to define the resolution of the

bioRxiv preprint doi: https://doi.org/10.1101/2025.05.06.652354; this version posted May 6, 2025. The copyright holder for this preprint (which was not certified by peer review) is the author/funder, who has granted bioRxiv a license to display the preprint in perpetuity. It is made available under aCC-BY 4.0 International license.

map in three dimensions and limit the influence of preferred particle orientation in the final map. (**B**) Density of the CDTb dimeric intermediate bound to CDTa colored to illustrate the location of CDTb Chain A (scarlet), CDTb Chain B (salmon), and CDTa (gold) is shown at the top. The fit of the generated model is shown for each chain below. (**C**) Residues predicted to be at the site of interaction between the CDTb dimer and CDTa. Residues interfacing with CDTb Chain A are shown in scarlet and residues interfacing with Chain B are shown in salmon. (**D**) A plot depicting the RMSD between the CDTb dimeric intermediate bound to CDTa and CDTa bound to the CDTb symmetric heptamer (PDB 6V1S). (**E**) Reconstruction of the CDTb trimeric intermediate in complex with CDTa led to the generation of a map resolved to 7.86 Å. (**F**) The relative orientations of Chains A (scarlet), B (salmon), and C (red) are illustrated for the apo CDTb trimeric intermediate (left), the trimer structure extracted from the CDTb symmetric heptamer (middle), and the CDTb trimeric assembly intermediate bound to CDTa (right). The relative orientations of Chains A, B, and C in the CDTb trimeric assembly intermediate more closely resemble that of the CDTb symmetric heptamer.

bioRxiv preprint doi: https://doi.org/10.1101/2025.05.06.652354; this version posted May 6, 2025. The copyright holder for this preprint (which was not certified by peer review) is the author/funder, who has granted bioRxiv a license to display the preprint in perpetuity. It is made available under aCC-BY 4.0 International license.

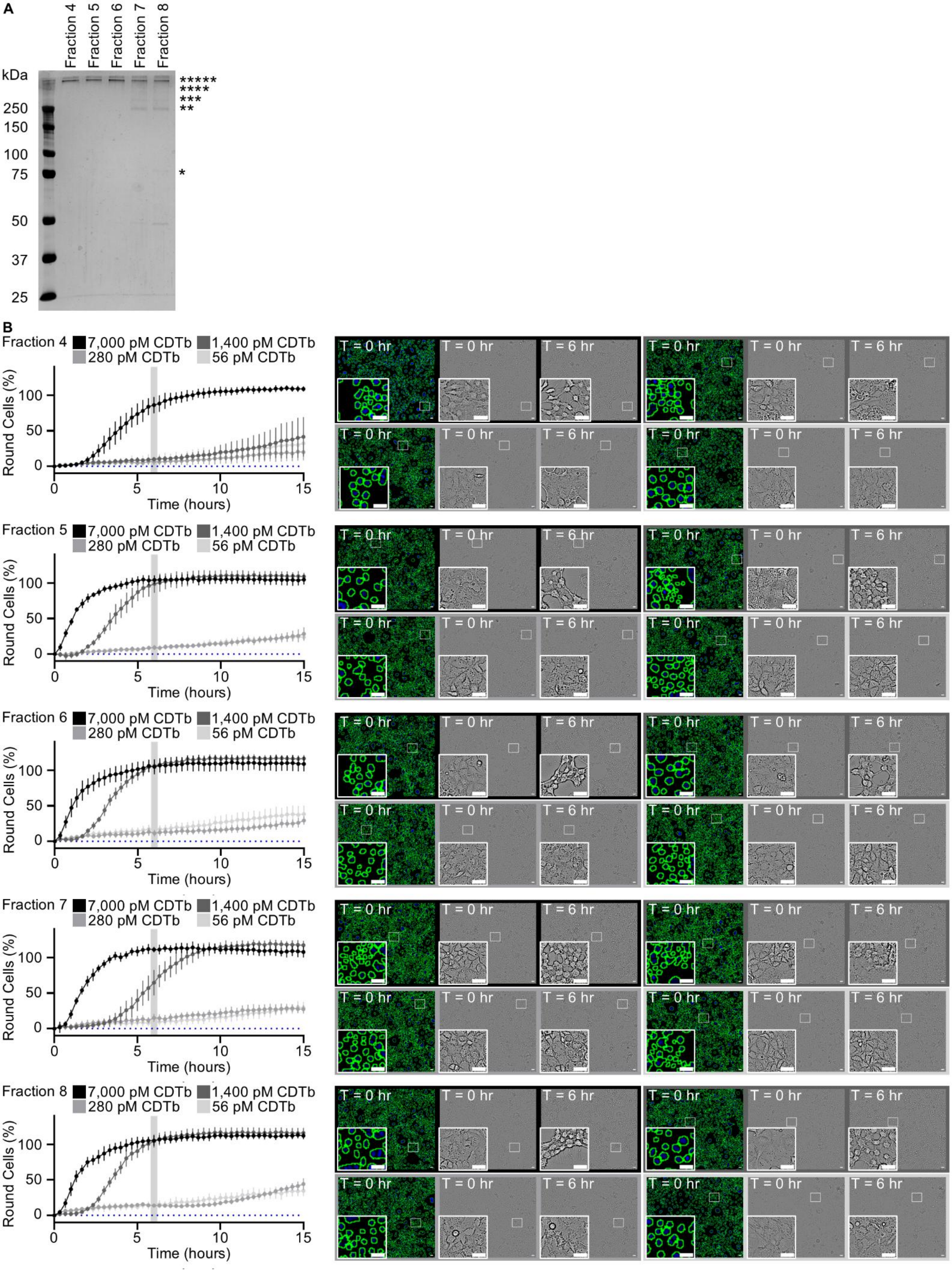

bioRxiv preprint doi: https://doi.org/10.1101/2025.05.06.652354; this version posted May 6, 2025. The copyright holder for this preprint (which was not certified by peer review) is the author/funder, who has granted bioRxiv a license to display the preprint in perpetuity. It is made available under aCC-BY 4.0 International license.

Supporting Fig 6 – Cellular Intoxication Assays Illustrating the Effectiveness of CDTb Assembly Intermediates. (**A**) CDTb assembly intermediates were isolated via size exclusion chromatography and analyzed by SDS-PAGE. A* indicates the CDTb monomer while **, ***, ****, and ***** indicate unique molecular species with molecular weights corresponding to oligomeric assembly intermediates. (**B**) Enumeration of cellular intoxication assays for all five fractions are shown for the entire fifteen-hour assay (left). Error bars represent standard deviation. Representative images for each concentration assayed are shown on the right with the panels color-coded to reflect the concentration as depicted in the graph on the left. The first image in each series depicts the enumeration of nuclei with blue indicating Hoechst staining and green indicating the computational assignment of a nucleus. The remaining two images in each sequence are bright field images that illustrate the number of round cells at the onset of the experiment (middle) and after a six-hour incubation (right).

bioRxiv preprint doi: https://doi.org/10.1101/2025.05.06.652354; this version posted May 6, 2025. The copyright holder for this preprint (which was not certified by peer review) is the author/funder, who has granted bioRxiv a license to display the preprint in perpetuity. It is made available under aCC-BY 4.0 International license.

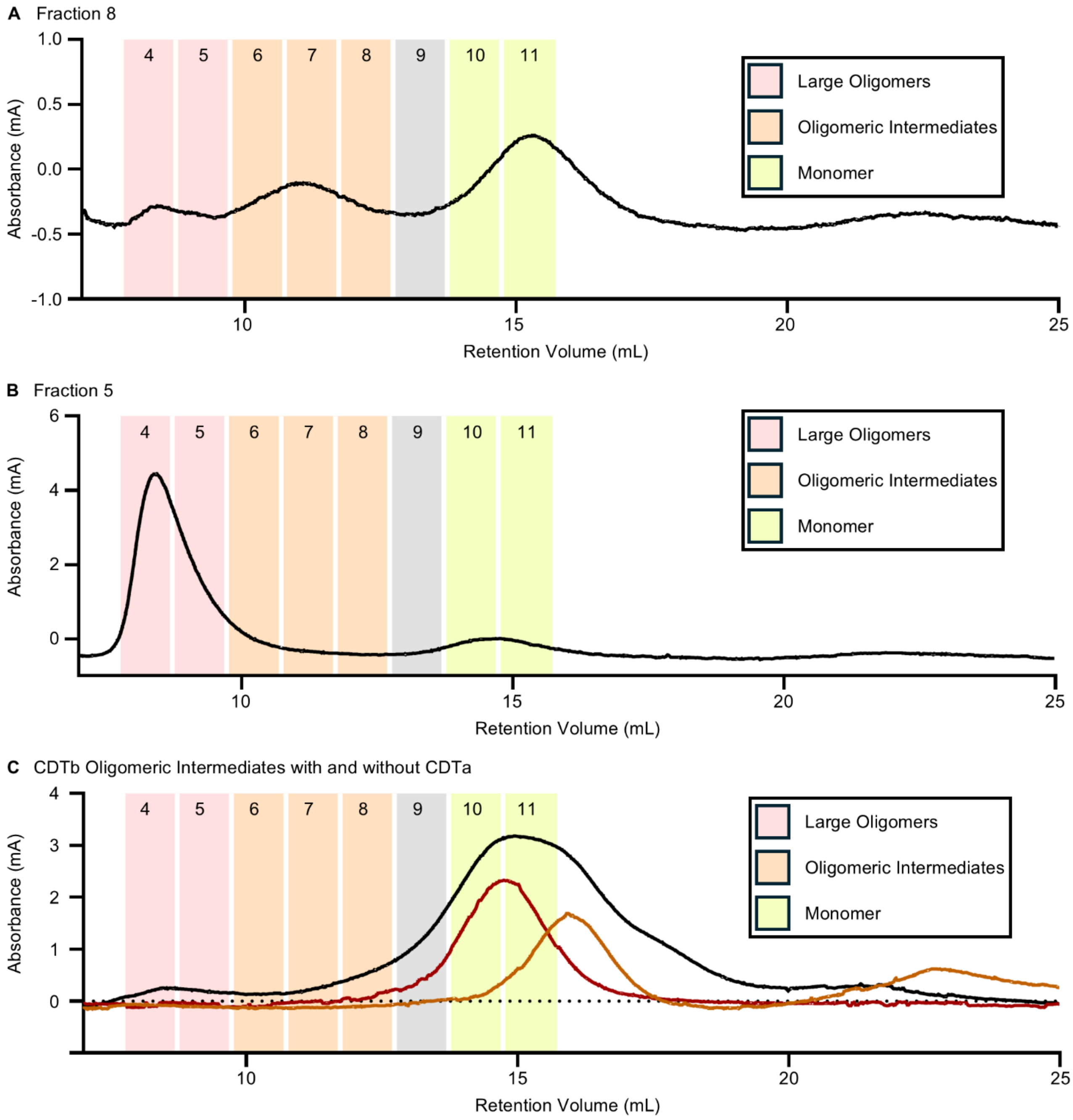

Supporting Fig 7 – CDTb Oligomeric Intermediate Assembly is Reversible. (**A**) The content of fraction eight was assessed via size exclusion chromatography and indicates the presence of large oligomeric particles (pink), oligomeric intermediates (orange), and the CDTb monomer (yellow). (**B**) The content of fraction five was assessed via size exclusion chromatography illustrating the presence of large oligomeric particles (pink) in this fraction. No oligomeric intermediates (orange) and a relatively low abundance of the CDTb monomer (yellow) were observed in this sample. (**C**) Size exclusion chromatography analysis of CDTa, CDTb oligomeric intermediates, and CDTb oligomeric intermediates in the presence of CDTa.

bioRxiv preprint doi: https://doi.org/10.1101/2025.05.06.652354; this version posted May 6, 2025. The copyright holder for this preprint (which was not certified by peer review) is the author/funder, who has granted bioRxiv a license to display the preprint in perpetuity. It is made available under aCC-BY 4.0 International license.

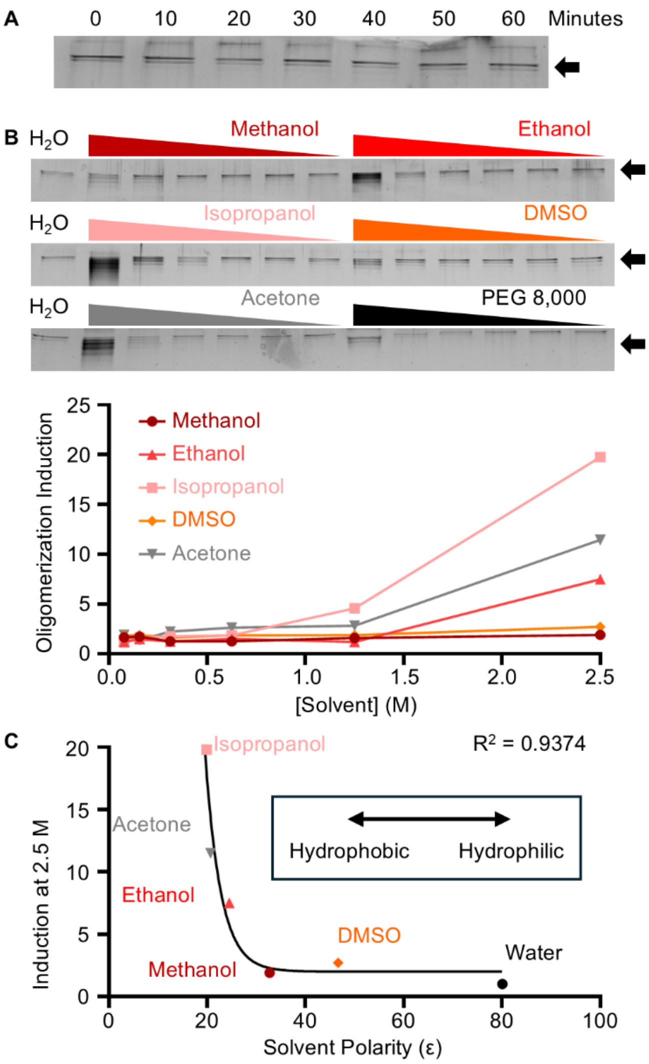

Supporting Fig 8 – Induction of CDTb Oligomerization by Hydrophobic Solvents. (**A**) In agreement with previous studies we note the oligomerization of CDTb is slow in vitro with little oligomer formation occurring after a 60-minute incubation. (**B**) Oligomerization was induced through the addition of various solvents and polyethylene glycol 8,000 (PEG 8,000). The concentration of the oligomer was quantified at various concentrations. DMSO – dimethyl sulfoxide. (**C**) The induction of oligomerization at 2.5 M solvent plotted against the polarity of the solvent and fit to an exponential decay function. DMSO – dimethyl sulfoxide.

Supporting Table 1 – Cryo-EM Data Collection and Refinement Statistics

**Data Collection and Processing**	**CDTb + CDTa**									
Voltage (kV)	300									
Electron dose ($e^{-}/Å^{2}$)	50									
Pixel size (Å)	0.86									
Defocus range	-0.5 to -1.5									
Number of Movies	6,344									
Total Particle Images	679,889									
**Map Reconstruction**	**CDTb Monomer**	**CDTb Dimer**		**CDTb Trimer**		**CDTb Tetramer**	**CDTb Pentamer**	**CDTb Hexamer**	**CDTb Heptamer**	**CDTb Heptamer (symmetric)**
EMDB Identifier	EMD-48170	EMD-48171	EMD-48172	EMD-48173	EMD-48174	EMD-48175	EMD-48176	EMD-48177		EMD-48178
Initial Particle Images	60,753	330,267		76,576		43,725	14,329	2,688	1,456	54,037
Apo/CDTa Bound	Apo	Apo	CDTa Bound	Apo	CDTa Bound	Apo	Apo	Apo	Apo	Apo
Initial Particle Images	60,753	156,814	173,453	72,140	4,436	43,725	14,329	2,688		39,547
Final Particle Images (no.)	51,697	78,323	21,744	43,047	4,436	25,019	14,329	2,688		39,547
Map resolution (Å)	6.83	3.56	5.17	4.19	7.86	7.01	10.32	12.05		3.33
Symmetry imposed	C1	C1	C1	C1	C1	C1	C1	C1		C7
FSC Threshold	0.143	0.143	0.143	0.143	0.143	0.143	0.143	0.143		0.143
**Refinement**										
PDB Identifier		9MDI	9MDJ	9MDL	9MDN	9MDP				9MDR
Model Resolution (Å)		3.5	5.2	4.1	8	6.8				3.3
FSC Threshold		0.143	0.143	0.143	0.143	0.143				0.143
Map Sharpening *B* Factor ($Å^{2}$)		165.8	204.5	196.5	356.9	611.4				157
Model Composition										
Non-Hydrogen Atoms		8,139	11,358	12,237	15,456	16,316				18,389
Protein Residues		1,043	1,440	1,569	1,966	2,092				2,352
Ligands		6	6	9	9	12				14
B factors ($Å^{2}$)										
Protein Residues		68.07	361.17	94.43	522.66	570.86				75.07
Ligands		70.81	254.82	100.11	326.40	493.13				52.39
**Validation**										
MolProbity Score		1.72	1.84	1.81	1.92	2.15				1.73
Clashscore		4.80	9.04	5.22	8.51	12.22				6.75
EMRinger Score		2.33	N/A*	0.64	N/A*	N/A*				2.31
Rotamer Outliers (%)		0.76	0.00	0.00	0.00	0.00				0.00
Ramachandran Plot										
Preferred (%)		92.4	94.9	90.8	92.7	90.0				94.9
Allowed (%)		7.6	5.10	9.2	7.3	10.0				5.1
Outliers (%)		0.0	0.0	0.0	0.0	0.0				0.0
Bond Deviation										
Lengths (Å)		0.004	0.004	0.003	0.003	0.004				0.003
Angles (°)		0.813	0.812	0.771	0.784	0.885				0.640

* EMRinger was not run for these datasets as it is not suitable for moderate to low resolution datasets.
